# HIV infection in adult Ugandans with new-onset type 2 diabetes: exploring its influence on the anthropometric and metabolic profile

**DOI:** 10.1186/s12981-023-00553-9

**Published:** 2023-08-18

**Authors:** Davis Kibirige, Isaac Sekitoleko, Noela Owarwo, Irene Andia-Biraro, William Lumu

**Affiliations:** 1 Department of Medicine, Uganda Martyrs Hospital Lubaga, Kampala, Uganda; 2 Non-communicable Diseases Program, Medical Research Council, Research Unit, Virus Research Institute and London School of Hygiene and Tropical Medicine Uganda, Entebbe, Uganda; 3 The Infectious Diseases Institute, College of Health Sciences, Makerere University Kampala, Kampala, Uganda; 4 Department of Internal Medicine, School of Medicine, College of Health Sciences, Makerere University, Kampala, Uganda; 5 Department of Medicine, Mengo Hospital, Kampala, Uganda

**Keywords:** Anthropometric characteristics, HIV infection, Insulin resistance, Metabolic profile, Pancreatic beta-cell function, Sub-Saharan Africa, Type 2 diabetes, Uganda

## Abstract

**Objective:**

HIV infection increases the risk of type 2 diabetes and may influence its phenotypic profile. In this study, we aimed to compare the anthropometric and metabolic characteristics of HIV-infected and uninfected adult Ugandans with new-onset type 2 diabetes to evaluate the influence of HIV infection on specific surrogate markers of adiposity, insulin resistance, and pancreatic beta-cell function.

**Methods:**

We consecutively recruited 500 HIV-infected and uninfected adult Ugandans with new-onset type 2 diabetes (diagnosed in < 3 months) from seven tertiary hospitals over a 20-month period and compared their anthropometric and metabolic characteristics to identify any significant differences.

**Results:**

Of the 500 participants with new-onset type 2 diabetes, 59 (11.8%) had a self-reported history of HIV infection. Compared with HIV-uninfected participants with type 2 diabetes, participants with HIV infection and type 2 diabetes had a lower median (IQR) hip circumference (97.8 [91.0-106.0] cm vs. 104.0 [96.0-112.0], p = 0.002) and visceral fat level (8 [6–11] vs. 10 [7–12], p < 0.001) assessed using bioimpedance analysis. No statistically significant difference was noted with the markers of pancreatic beta-cell function (fasting, 30-minute, and 120-minute C-peptide concentrations, oral insulinogenic index, and homeostatic model assessment 2-beta cell function) and insulin resistance (homeostatic model assessment 2-insulin resistance) between both groups.

**Conclusion:**

In our study population, HIV infection was not associated with increased adiposity, pancreatic beta-cell function, and insulin resistance. Large prospective studies are needed to investigate the effect of HIV on the pathogenesis of type 2 diabetes in adult Ugandans.

## Introduction

Sub-Saharan Africa (SSA), including Uganda, is currently experiencing a steadily growing double burden of HIV infection and type 2 diabetes (T2D) [1, 2]. The most recently documented prevalence of HIV infection and T2D in Uganda is 5.5% and 1.4%, respectively [3, 4].

Compelling evidence shows that HIV infection directly or indirectly increases the risk of developing T2D. Specific sociodemographic, clinical, and metabolic factors associated with HIV infection like increasing age, the effect of antiretroviral therapy (ART) like dolutegravir and protease inhibitors, chronic low-grade inflammation, lipodystrophy, and metabolic syndrome are some of the reasons to explain this well-documented increase in the risk [5–9].

Type 2 diabetes is characterised by marked heterogeneity in clinical presentation and pathophysiology [10]. Several underlying pathophysiologic defects, including insulin resistance, pancreatic beta-cell dysfunction, increased lipolysis, and reduced incretin effect, have been suggested to explain the observed hyperglycaemia in T2D [11].

Studies that have investigated the influence of HIV infection on the onset of T2D in African patients have reported inconsistent results. In one systematic review and meta-analysis of 20 studies that assessed the incidence and prevalence of T2D in African HIV-infected patients, no significant difference was noted in the prevalence of T2D in HIV-infected and uninfected individuals. However, the majority of these studies were small with high heterogeneity and moderate to high risk of bias as a key limitation [12]. However, in another systematic review and meta-analysis by Mulindwa et al. that included 16 studies assessing the association between the use of integrase strand transfer inhibitors (INSTI), insulin resistance, and incident T2D, the two studies conducted in African HIV-infected individuals reported a threefold increase in the risk of new-onset T2D. However, these two studies did not assess the effect of INSTI on insulin resistance [9].

With the increased roll-out and improved access to ART in SSA, including Uganda, the life expectancy of HIV-infected individuals has increased with more patients with non-communicable diseases like T2D and hypertension. Studies that have robustly assessed the influence of HIV infection, either directly or indirectly via the effects of ART, on some of the above pathophysiologic processes in adult Africans with T2D are limited. Understanding its influence on the pathophysiologic processes would be key in informing appropriate therapeutic and preventive strategies for T2D in this patient population, in addition to understanding its effect on the optimal control of the specific metabolic parameters like glycated haemoglobin and lipid profile.

As part of the Uganda Diabetes Phenotype (UDIP) study that investigated the manifestation of diabetes in adult Ugandan patients, the objective of this sub-study was to compare specific surrogate markers of adiposity (body mass index or BMI, waist- and hip circumference, visceral fat levels), insulin resistance (homeostatic model assessment-2 insulin resistance or HOMA2-IR) and pancreatic beta-cell function (homeostatic model assessment-2-beta cell function or HOMA2-%B, fasting C-peptide, oral insulinogenic index or IGI) between HIV-infected and uninfected adult Ugandans with new-onset T2D to fully understand the role of HIV infection in the pathophysiology of T2D in this study population and to guide individualised diabetes therapy.

## Methods

The UDIP study was a cross-sectional study that consecutively enrolled adult participants (aged ≥ 18 years) presenting with recently diagnosed T2D (diagnosis made in < 3 months without evidence of pancreatic beta-cell autoimmunity) at different diabetes outpatient clinics. The diagnosis of T2D was made by the attending healthcare workers following by the World Health Organisation guideline on the diagnosis of diabetes [13]. Both treatment naïve and patients on glucose-lowering therapy were included. We excluded pregnant women with new-onset diabetes.

We consecutively recruited 59 participants with a self-reported diagnosis of HIV infection and 441 HIV-uninfected adult participants with recently diagnosed confirmed T2D from seven public and faith-based private not-for-profit tertiary hospitals located in Central and Southwestern Uganda between February 2019 and October 2020.

Using standardised study procedures and pre-tested study questionnaire, we collected relevant information on the sociodemographic (age and sex) and anthropometric (weight, height, body mass index or BMI, waist circumference or WC hip circumference or HC, and waist: hip circumference ratio or WHR) characteristics from all participants. Weight and height measurements were performed using a Seca® digital weighing scale and stadiometer, respectively. We also assessed the visceral fat levels indirectly by bioimpedance analysis (BIA) using an OMRON BF511 body composition monitor (Omron® Healthcare, Tokyo, Japan).

All participants had a fasting venous blood sample drawn for the measurement of glycated haemoglobin (HbA1c), fasting blood glucose (FBG), C-peptide, and insulin concentrations followed by a 75-gram oral glucose tolerance test (OGTT) for measurement of the 30- and 120-minute C-peptide concentrations and calculation of the oral insulinogenic index (IGI), as the surrogate markers of pancreatic beta-cell secretory function. The homeostatic model assessment-2 (HOMA2) insulin resistance (HOMA2-IR) and HOMA2-beta cell function (HOMA2-%B), as surrogate markers of insulin resistance and pancreatic beta-cell function respectively, were assessed using the online HOMA2 calculator [14].

All the above tests were carried out at the Medical Research Council/Uganda Virus Research Institute and London School of Hygiene and Tropical Medicine Uganda Research Unit, Entebbe Uganda. The HbA1c, lipid profile, C-peptide, and insulin tests were conducted using electro-chemiluminescence immunoassays manufactured by Roche diagnostics Limited, Germany on a Cobas 6000 C-model SN 14H3-15 machine (Hitachi High Technologies Corporation, Tokyo Japan). The fasting blood glucose concentration was determined quantitatively using the hexokinase enzymatic principle using the same Cobas 6000 machine.

### Statistical analysis

The categorical and continuous variables describing all the study participants were expressed as proportions and medians with inter-quartile range (IQR), respectively. The differences in the socio-demographic and metabolic characteristics between the HIV-infected and uninfected participants with recently diagnosed T2D were analysed using the chi-square test for categorical data and the Kruskal Wallis test for continuous data. The latter test was preferred because the data wasn’t uniformly distributed.

All analyses were done using STATA statistical software version 15 College Station, TX: StataCorp LLC. A p-value < 0.05 was considered statistically significant.

## Results

### Baseline characteristics of all study participants

Table 1 summarises the demographic, anthropometric, and metabolic characteristics of all participants and the HIV-infected and uninfected participants with recently diagnosed T2D.

**Table 1:** Socio-demographic, clinical, anthropometric, and metabolic characteristics of all study participants, and those with and without HIV infection and type 2 diabetes morbidity

Characteristics	All study participants (n = 500)	HIV-infected participants with T2D (n = 59, 11.8%)	HIV-uninfected participants with T2D (n = 441, 88.2%)	P-value
Age, years	48 (39–58)	53 (46–57)	47 (39–58)	0.01
Females	283 (56.6)	37 (62.7)	246 (55.8)	0.31
**Markers of adiposity** BMI, kg/m^2^	27.4 (23.6–31.4)	25.8 (23.3–29.6)	27.7 (23.6–31.6)	0.06
WC, cm	96.0 (87.0-104.8)	94.0 (86.0-101.5)	96.0 (87.5–105.0)	0.42
HC, cm	103.0 (96.0-111.5)	97.8 (91.0-106.0)	104.0 (96.0-112.0)	0.002
WHR	0.92 (0.88–0.96)	0.95 (0.91–0.99)	0.92 (0.87–0.96)	0.007
Visceral fat level on BIA	9 (7–12)	8 (6–11)	10 (7–12)	< 0.001
**Metabolic markers** HbA1c, %	10.3 (7.7–12.5)	11.3 (8.5–12.7)	10.2 (7.7–12.5)	0.16
HbA1c, mmol/mol	90 (61–113)	100 (69–115)	88 (60–113)	0.16
Fasting blood glucose, mmol/l	8.6 (6.2–13.4)	9.0 (5.8–14.8)	8.6 (6.2–13.1)	0.58
Total cholesterol, mmol/l	4.0 (3.3-5.0)	3.9 (3.2–5.1)	4.1 (3.3-5.0)	0.78
High-density lipoprotein cholesterol, mmol/l	0.95 (0.74–1.20)	0.89 (0.70–1.20)	0.96 (0.75–1.20)	0.26
Triglycerides, mmol/l	1.3 (1.0-1.8)	1.5 (1.1–2.1)	1.3 (1.0-1.8)	0.06
Low-density lipoprotein cholesterol, mmol/l	2.6 (1.9–3.4)	2.4 (1.9–3.3)	2.6 (1.9–3.4)	0.31
Non-high-density lipoprotein cholesterol, mmol/l	3.0 (2.4–3.8)	2.9 (2.4–3.9)	3.0 (2.4–3.8)	0.80
**Markers of pancreatic** **beta-cell function** Fasting serum C-peptide, ng/ml	1.4 (0.8–2.1)	1.5 (1.0–2.0)	1.4 (0.8–2.1)	0.50
30-minute serum C-peptide, ng/ml (post-OGTT)	2.1 (1.1–3.3)	2.1 (1.5-3.0)	2.1 (1.1–3.4)	0.94
120-minute serum C-peptide, ng/ml (post-OGTT)	2.8 (1.5–4.8)	2.7 (1.6-4.0)	2.9 (1.5–4.9)	0.77
Fasting insulin, µU/ml	5.9 (3.0-10.6)	6.7 (4.2–9.8)	5.8 (2.9–10.7)	0.35
Oral IGI, µU/mmol	1.31 (0.47–3.85)	1.06 (0.40–3.56)	1.38 (0.48–3.96)	0.48
HOMA2- %B	43.1 (20.7–77.6)	39.2 (15.4–77.8)	43.5 (22.3–77.2)	0.85
**Marker of insulin resistance** HOMA2-IR	1.21 (0.77–2.03)	1.24 (0.82–2.11)	1.20 (0.76–2.01)	0.83

The categorical and continuous variables are presented as percentages and median (interquartile ranges), respectively

The median (IQR) age, HbA1c, and C-peptide of all participants was 48 (39–58) years, 10.3 (7.7–12.5) %, and 1.4 (0.8–2.1) ng/ml, respectively. About 57% of the participants were female. Of the 500 participants with recently diagnosed T2D, 59 participants (11.8%) had a self-reported history of HIV infection and all were on a tenofovir-lamivudine-dolutegravir combination which is the first-line ART recommended by the HIV treatment guidelines of the Ministry of Health, Republic of Uganda.

### Comparison of the anthropometric and metabolic characteristics between the HIV-infected and uninfected participants with recently diagnosed type 2 diabetes

Considering specific markers of adiposity that are closely related to insulin resistance, compared with those who were HIV-uninfected with recently diagnosed T2D, participants with HIV infection and T2D comorbidity had lower median (IQR) HC (97.8 [91.0-106.0] cm vs. 104.0 [96.0-112.0] cm, p = 0.002) and visceral fat levels on BIA (8 [6–11] vs. 10 [7–12], p < 0.001), and a higher median (IQR) WHR (0.95 [0.91–0.99] vs. 0.92 [0.87–0.96], p = 0.007).

Regarding all the markers of pancreatic beta-cell function, there were no significant differences noted in the fasting, 30-minute, and 120-minute C-peptide concentrations, the oral IGI, and HOMA2-%B between the HIV-infected and uninfected participants with recently diagnosed T2D. We also did not observe any statistically significant differences in the HOMA2-IR as a surrogate marker of insulin resistance between both groups (1.24 [0.82–2.11] vs. 1.20 [0.76–2.01], p = 0.83).

## Discussion

In this study population, we demonstrated that HIV infection was associated with lower markers of adiposity and did not have an effect on insulin resistance and pancreatic beta-cell function.

Our finding of lower markers of adiposity in the HIV-infected participants with recently diagnosed T2D is contrary to what has been reported in the literature reporting a high frequency of obesity and visceral adiposity associated with HIV infection mainly due to the effect of ART [15, 16]. This difference in the markers of adiposity reflect the heterogeneity in populations and may also be explained by the differences in environmental and genetic factors.

Despite the well-described effects of chronic low-grade inflammation, immune dysregulation, adipose tissue dysfunction, and effects of specific ART of resulting in increased insulin resistance, an atherogenic lipid profile, and pancreatic beta-cell dysfunction [17–20], we did not observe increased insulin resistance, beta-cell secretory dysfunction, and an abnormal lipid profile in our HIV-infected study population.

A similar finding of a lack of an influence of HIV infection on the markers of insulin resistance and pancreatic beta-cell function was also reported in one study called the Chronic Infections, Comorbidities and Diabetes in Africa (CICADA) study that investigated the risk factors of diabetes in HIV-uninfected and HIV-infected adults in North-western Tanzania. No association between HIV infection and oral IGI, as a surrogate marker of pancreatic beta-cell function, was reported in this study [21].

In another study conducted on 710 HIV-infected and 226 HIV-uninfected Rwandan women, compared with those who were HIV-uninfected, HIV-infected participants had a lower mean HOMA2-IR level. It is important to note that all of these participants were ART naïve and had a low baseline mean BMI of 21.3 ± 3.7 kg/m^2^, and this could explain the study findings [22]. Conversely, HIV infection has been reported to be associated with a high prevalence of insulin resistance in some studies conducted in sub-Saharan African HIV-infected patients, especially in females and in the presence of obesity and poor virological suppression [23–25].

The lack of an influence of HIV infection on the pancreatic beta-cell function and insulin resistance in our study population suggests that the underlying pathophysiologic defects of T2D in adult Ugandans may be explained by other environmental exposures like early-onset malnutrition, tropical infections like malaria, and genetic influences with minimal impact of HIV infection.

Our study had some strengths. Because we recruited adult participants with a recent diagnosis of diabetes (a diagnosis made in the preceding three months), this reduced the confounding effect of long-standing diabetes on the phenotypic characteristics being investigated (markers of pancreatic beta-cell function and insulin resistance). We also performed several laboratory tests to evaluate the pancreatic beta-cell function and insulin resistance.

Our study had some limitations. Because of its cross-sectional design, we are unable to make any causal inferences. We did not collect information on the extent of viral suppression and immunological status (viral load count and CD4 count), opportunistic infections (current and past), and duration of HIV infection and ART use. These factors could further help explain our study findings. The study participants were mainly recruited from tertiary hospitals located in central Uganda, hence we cannot generalise the study findings to the entire Ugandan population.

## Conclusion

In this study, we demonstrated that HIV infection is not associated with increased adiposity, insulin resistance, and pancreatic beta-cell dysfunction in adult Ugandans with recently diagnosed T2D. This study findings signifies that other environmental factors and/or genetic influences, other than HIV infection, may play a more significant role in the onset of T2D in the adult Ugandan population.

However, large well-designed prospective studies are needed to robustly investigate the effect of HIV infection, specific ART, degree of virological suppression, and immunological status on adiposity, insulin resistance, pancreatic beta-cell function, and metabolic control in adult Ugandans with T2D and further confirm these findings.

## Data Availability

The datasets used and/or analysed during the current study are available from the corresponding author on reasonable request.

## List of abbreviations

ART: Antiretroviral therapy
BIA: Bioimpedance analysis
BMI: Body mass index
CICADA: Chronic Infections Comorbidities and Diabetes in Africa
FBG: Fasting blood glucose
HbA1c: glycated haemoglobin
HC: Hip circumference
HOMA2-IR: Homeostatic model assessment-2 insulin resistance
HOMA2-%B: Homeostatic model assessment-2 beta cell function
IGI: Insulinogenic index
IQR: Inter-quartile range
OGTT: Oral glucose tolerance test
T2D: Type 2 diabetes
UDIP: Uganda Diabetes Phenotype
WC: Waist circumference
